# Early Origin and Evolution of the Angelman Syndrome Ubiquitin Ligase Gene *Ube3a*

**DOI:** 10.3389/fncel.2017.00062

**Published:** 2017-03-07

**Authors:** Masaaki Sato

**Affiliations:** ^1^Graduate School of Science and Engineering and Brain and Body System Science Institute, Saitama UniversitySaitama, Japan; ^2^RIKEN Brain Science InstituteWako, Japan

**Keywords:** brain evolution, genomic imprinting, developmental disorder, autism, intellectual disability, HECT domain, synapse

## Abstract

The human *Ube3a* gene encodes an E3 ubiquitin ligase and exhibits brain-specific genomic imprinting. Genetic abnormalities that affect the maternal copy of this gene cause the neurodevelopmental disorder Angelman syndrome (AS), which is characterized by severe mental retardation, speech impairment, seizure, ataxia and some unique behavioral phenotypes. In this review article, I highlight the evolution of the *Ube3a* gene and its imprinting to provide evolutionary insights into AS. Recent comparative genomic studies have revealed that *Ube3a* is most phylogenetically similar to *HECTD2* among the human HECT (homologous to the E6AP carboxyl terminus) family of E3 ubiquitin ligases, and its distant evolutionary origin can be traced to common ancestors of fungi and animals. Moreover, a gene more similar to *Ube3a* than *HECTD2* is found in a range of eukaryotes from amoebozoans to basal metazoans, but is lost in later lineages. Unlike in mice and humans, *Ube3a* expression is biallelic in birds, monotremes, marsupials and insects. The imprinting domain that governs maternal expression of *Ube3a* was formed from non-imprinted elements following multiple chromosomal rearrangements after diversification of marsupials and placental mammals. Hence, the evolutionary origins of *Ube3a* date from long before the emergence of the nervous system, although its imprinted expression was acquired relatively recently. These observations suggest that exogenous expression and functional analyses of ancient *Ube3a* orthologs in mammalian neurons will facilitate the evolutionary understanding of AS.

## Introduction

The evolution of the brain in mammals is characterized by dramatic increases in size and complexity, especially in the neocortex (Striedter, 2005). Previous advances in comparative genomics have revealed emerging principles of the genetic basis of brain evolution (Khaitovich et al., 2006; Vallender et al., 2008). Changes in protein-coding sequences and regulatory elements as well as emergence of new genes and loss of existing ones likely had profound phenotypic impacts on brain development and ultimately led to significant alterations in brain structure and function. Thus, understanding how genes that play essential roles in human brain development and cognition evolved is of great importance and interest.

The ubiquitin ligase gene *Ube3a* (also known as E6-associated protein; *E6AP*) provides an excellent model for studies of gene evolution because of its brain-specific imprinting and implication in the neurodevelopmental disorder Angelman syndrome (AS). Ube3a is a homologous to the E6AP carboxyl terminus (HECT) domain-containing E3 ubiquitin ligase that was initially discovered as the protein involved in human papillomavirus E6-mediated p53 degradation (Huibregtse et al., 1993). It is expressed monoallelically from the maternal allele in the brain in a parent-of-origin specific manner (Albrecht et al., 1997; Rougeulle et al., 1997; Vu and Hoffman, 1997). The imprinting of *Ube3a* and its neighboring genes is coordinated by a regulatory region known as the Prader-Willi syndrome (PWS)-AS imprinting center (IC), which is located upstream of the adjacent *SNURF* (SNRPN upstream reading frame)–*SNRPN* (small nuclear ribonucleoprotein-associated protein N) gene on the human 15q11-q13 chromosome region (Buiting et al., 1999; Ohta et al., 1999; Perk et al., 2002). Genetic abnormalities that affect the maternal copy of *Ube3a* are known to cause AS, which is characterized by a wide variety of symptoms such as severe mental retardation, speech impairment, seizure, ataxia and unique behavioral phenotypes such as frequent laughter (Angelman, 1965; Williams et al., 1995; Kishino et al., 1997; Matsuura et al., 1997; Clayton-Smith and Laan, 2003; Mabb et al., 2011; Buiting et al., 2016), whereas duplication or increased expression of this gene is linked to autism spectrum disorders (Bolton et al., 2001; Glessner et al., 2009; Smith et al., 2011; Urraca et al., 2013). Accordingly, *Ube3a* is essential for neural circuit maturation and experience-dependent plasticity in the mammalian cerebral cortex (Yashiro et al., 2009; Sato and Stryker, 2010). In this review article, I highlight the evolution of the *Ube3a* gene and its imprinting to gain evolutionary insights into AS.

## Ancient Origin of the *Ube3a* Gene

**Figure 1:**
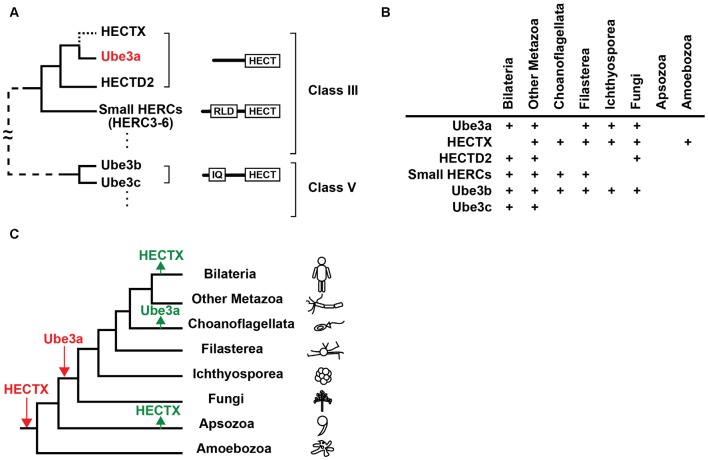
**Evolutionary origin of Ube3a. (A)** Phylogenetic relationships, domain structures, and classes of homologous to the E6AP carboxyl terminus (HECT) E3 ligases that are similar to Ube3a. The branch to HECTX that was lost in bilaterians is indicated by a dotted line. For a simplified diagram, branches that connect class III and class V E3 ligases are shown with a dashed line. IQ, IQ domain; RLD, RCC1-like domain. **(B)** Presence of orthologs of Ube3a, HECTX, HECTD2, small HERCs, Ube3b and Ube3c in eukaryotes. **(C)** Gains (red) and losses (green) of Ube3a and HECTX are overlaid on a eukaryotic tree of life. Figures were modified from Grau-Bové et al. (2013).

Ube3a contains a single HECT domain at the C-terminal and no discernible functional domain at its N-terminal side. Recent analyses revealed that the Ube3a protein is phylogenetically closest to HECTD2 among 28 human HECT domain-containing ubiquitin ligases, and a group of proteins called small HERCs (HERC3-6) that possess a single N-terminal RCC1-like domain (RLD) and a C-terminal HECT domain are also similar to Ube3a (Marín, 2010; Grau-Bové et al., 2013; Scheffner and Kumar, 2014; Figure 1A). HECTD2 and small HERCs are expressed in the brain, although their functions are not well understood (Sánchez-Tena et al., 2016). *HECTD2* has been associated with susceptibility to neurological diseases (Lloyd et al., 2009a,b). Two other HECT E3 ligases, Ube3b and Ube3c, were named after Ube3a (Gong et al., 2003), but carry a calmodulin-binding IQ domain in addition to a HECT domain and are categorized as a distinct class of HECT E3 ligases (Figure 1A). Notably, *Ube3b* has been strongly implicated in the human developmental disorder blepharophimosis-ptosis-intellectual-disability syndrome (Basel-Vanagaite et al., 2012).

Orthologs of *Ube3a* are commonly found in bilaterians including vertebrates and *Drosophila* (Reiter et al., 2006; Wu et al., 2008; Hope et al., 2016) but not in *Caenorhabditis elegans*, implying that *Ube3a* was lost in some nematode lineages (Marín, 2010). The evolutionary origins of *Ube3a* can be traced to the genomes of basal metazoans, including the cnidarian *Nematostella vectensis*, the sponge *Amphimedon queenslandica*, and the placozoan *Trichoplax adherens* (Marín, 2010), and to fungi such as *Mortierella verticillata* (Grau-Bové et al., 2013; Figures 1B,C). However, no *Ube3a* orthologs are present in choanoflagellates, suggesting secondary loss in this lineage (Grau-Bové et al., 2013). Notably, the E3 ligase gene *HECTX* is more similar to *Ube3a* than *HECTD2* and is found in the genomes of amoebozoans, fungi, choanoflagellates and early metazoans, but not in those of bilaterians (Marín, 2010; Grau-Bové et al., 2013; Figures 1A–C). These findings suggest that an ancient E3 ligase gene that was more similar to *Ube3a* than the extant *HECTD2* was secondarily lost in the bilaterian lineage.

The evolution of Ube3a substrate specificity remains unclear. Several neuronal proteins have been identified to date as possible direct ubiquitination targets of Ube3a (Sell and Margolis, 2015; Sun et al., 2015). Although the precise modes of these protein interactions have not been characterized, specific substrate recognition by Ube3a is thought to be mediated by its non-catalytic N-terminal region (Cooper et al., 2004; Scheffner and Kumar, 2014). Whereas Ube3a orthologs share the conserved HECT domain at their C-termini, their N-terminal regions are more variable among lineages, suggesting that ancient Ube3a orthologs recognized differing sets of target proteins to those ubiquitinated by the present human Ube3a. New substrate specificity was likely acquired during evolution by changes in substrate binding regions and encounters of Ube3a with potential novel substrates, the latter of which were probably brought by changes in expression and subcellular localization, and the emergence of new proteins.

## *Ube3a* and the Evolution of the Nervous System

The early origin of* Ube3a* indicates that it predates the origins of nerve cells and synapses. The expression and function of the Ube3a protein in primitive organisms are currently unclear. Whether the nervous system evolved from single or multiple independent origins remains controversial (Miller, 2009; Ryan et al., 2013; Moroz et al., 2014; Liebeskind et al., 2016). At the base of the metazoan tree, sponges and placozoans lack nerve and muscle cells, but exhibit coordinated behaviors such as feeding and contraction (Ellwanger et al., 2007; Smith et al., 2015). On the other hand, cnidarians and ctenophores have diffuse nervous systems called nerve nets, which communicate by synapses (Anderson and Spencer, 1989; Tamm and Tamm, 1995; Marlow et al., 2009). Centralized nervous systems evolved in the bilaterian lineage (Arendt et al., 2016).

Searches for orthologs of specific postsynaptic density (PSD) proteins demonstrate that the genomes of nerve-less basal metazoans and unicellular choanoflagellates contain core sets of scaffold protein orthologs, and these are co-expressed in a distinct cell type of *Amphimedon* larvae (Sakarya et al., 2007; Alié and Manuel, 2010). Shank postsynaptic scaffold proteins have been implicated in autism spectrum disorders in humans (Durand et al., 2007; Berkel et al., 2010; Sato et al., 2012) and are also found in the choanoflagellate genome (Alié and Manuel, 2010), providing another remarkable example of the ancient origins of genes that are involved in human developmental disorders. More recent studies showed that human PSD proteins that are essential for basic cellular processes, such as amino acid biosynthesis and energy generation, are conserved between prokaryotes and eukaryotes, whereas the majority of structural and signaling molecules, including those involved in ubiquitination, are specific to eukaryotes (Emes and Grant, 2011). The ancient eukaryotic origin of *Ube3a* is thus consistent with the early origins of human postsynaptic proteins, many of which are linked to neurogenetic disorders (Bayés et al., 2011).

## Assembly of the PWS-AS Imprinted Domain

*Ube3a* expression is imprinted in the brain but not in peripheral tissues in humans and mice (Albrecht et al., 1997; Rougeulle et al., 1997; Vu and Hoffman, 1997). Moreover, *Ube3a* expression is imprinted in neurons but not in glial cells of the brain (Yamasaki et al., 2003; Judson et al., 2014). Interestingly, imprinting of *Ube3a* is not fully established in the postnatal mouse brain and paternal *Ube3a* expression decreases as neurons mature (Sato and Stryker, 2010; Judson et al., 2014). Hence, imprinted expression of *Ube3a* is tissue- and cell type-specific and is developmentally regulated.

Genomic imprinting, or parent-of-origin specific epigenetic gene silencing, is widespread in placental mammals and also occurs in marsupials, suggesting evolution from common ancestors of marsupials and eutherians (Renfree et al., 2009). Genome-wide characterization of imprinted genes revealed parent-of-origin allelic effects in over 1300 loci in embryonic and adult mouse brains (Gregg et al., 2010). However, hypotheses regarding the origins and evolutionary advantages of genomic imprinting are few. Among these, the host defense hypothesis proposes that genomic imprinting evolved from the cellular mechanisms that mediate methylation and silencing of foreign DNA elements (Barlow, 1993). Alternatively, the kinship theory suggests fitness advantages of genomic imprinting. Specifically, paternally and maternally expressed genes have been shown to increase and decrease the transfer of maternal nutrients to the fetus during pregnancy, respectively, as observed for the paternally expressed *IGF2* growth factor and the maternally expressed IGF2 receptor (*IGF2R*) growth repressor (Haig, 2004). Another hypothesis, the coadaptation theory, proposes that genomic imprinting coordinates placental and hypothalamic functions of the fetus and mother to optimize growth, postnatal suckling and maternal care, as demonstrated by the paternal *Peg3* transcription factor that is expressed in these tissues (Li et al., 1999; Curley et al., 2004).

**Figure 2:**
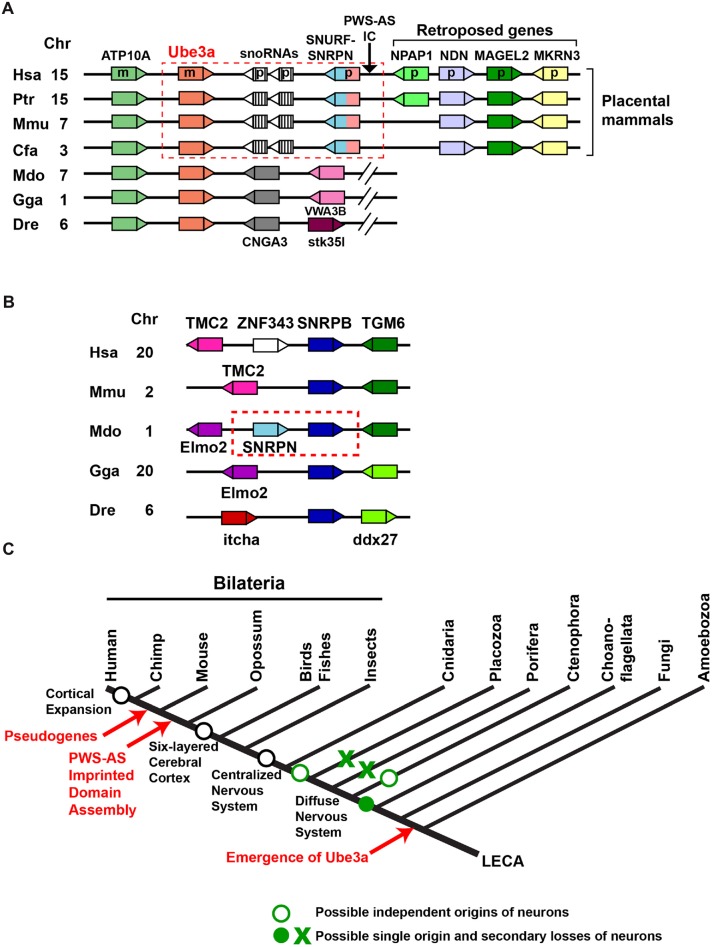
**Assembly of the imprinted *Ube3a* locus in the genomes of placental mammals. (A)** A schematic representation of arrangements of *Ube3a* in vertebrate genomes. Genes are represented as boxes, and triangles indicate their transcriptional directions. The same colors indicate the same genes across species. The conserved arrangement of *Ube3a* and SNRPN upstream reading frame (*SNURF*)*–*small nuclear ribonucleoprotein-associated protein N (*SNRPN*) in placental mammals is enclosed by a red dashed box. The arrow indicates the chromosomal position of the Prader-Willi syndrome-Angelman syndrome imprinting center (PWS-AS IC) located upstream of the *SNURF–SNRPN* gene in placental mammals. Hsa, *Homo sapiens*; Ptr, *Pan troglodytes*; Mmu, *Mus musculus*; Cfa, *Canis lupus familiaris*; Mdo, *Monodelphis domestica*; Gga, *Gallus gallus*; Dre, *Danio rerio*; Chr, chromosomal location in each species; m, maternally expressed gene in humans; p, paternally expressed gene in humans. **(B)** A schematic representation of the arrangement of *SNRPB* in vertebrate genomes. The tandem duplication of *SNRPN* from *SNRPB* in the opossum genome is enclosed by a red dashed box. **(C)** Early origin and evolution of *Ube3a* in relation to the evolution of the nervous system. LECA, last eukaryotic common ancestor.

A comparative genomic study revealed an unexpected picture of the assembly of the PWS-AS imprinted domain during evolution (Rapkins et al., 2006). In human chromosome 15q and homologous mouse chromosome 7C regions, *Ube3a* is located downstream of *SNURF–SNRPN*, which forms a bicistronic transcript and is expressed from the paternal allele. Maternal expression of *Ube3a* and paternal expression of *SNURF–SNRPN* are controlled by the IC that lies upstream of *SNURF–SNRPN* (Figure 2A). This arrangement is conserved in eutherians including mice and humans, but is not present in marsupials such as the gray short-tailed opossum *Monodelphis domestica* and other animals of greater evolutionary age. In these animals, the gene *CNGA3* is present downstream of *Ube3a* instead of *SNURF–SNRPN* (Figure 2A). Accordingly, expression of *Ube3a* is biallelic in the marsupial tammar wallaby, the montreme platypus, and in chickens and *Drosophila* (Colosi et al., 2006; Rapkins et al., 2006; Hope et al., 2016). The searches for the marsupial ortholog of *SNRPN* revealed that it resides beside the closely related *SNRPB* gene in the *Monodelphis domestica* genome (Figure 2B). Furthermore, the genomes of evolutionarily older animals including monotremes have *SNRPB* but no *SNRPN* orthologs. These findings suggest that *SNRPN* was formed by tandem duplication of the evolutionally older *SNRPB* gene in marsupials.

The PWS-AS imprinted domain encompasses a genomic region of about 2 Mb in humans and comprises a smaller AS subdomain that includes two maternally expressed genes (*Ube3a* and *ATP10A*) and a larger PWS subdomain that contains six paternally expressed genes (*MKRN3, MAGEL2, NDN, NPAP1, SNURF* and* SNRPN*) and two major clusters of the paternally expressed small nucleolar RNAs (snoRNAs) SNORD115 (HBII-52) and SNORD116 (HBII-85). Numbers of snoRNA genes in this region are highly variable across eutherian lineages (Zhang et al., 2014). In addition, the paternally expressed *NPAP1* is primate specific and lacks an ortholog in other eutherians (Neumann et al., 2014), suggesting that this imprinted domain is a highly dynamic genomic region. Currently, it is held that the PWS-AS imprinted domain formed from non-imprinted components by genome rearrangement in an eutherian ancestor after divergence from marsupials. The following ordered or concurrent steps have been proposed (Hore et al., 2007; Renfree et al., 2009): (1) fission of *Ube3a-CNGA3* border; (2) translocation of *SNRPN* next to *Ube3a*; (3) generation of *SNURF* and IC; (4) insertion and expansion of snoRNA repeats; (5) insertion of the three retroposed genes *MKRN3*, *MAGEL2*, and *NDN*, followed by the integration of *NPAP1* in the primate lineage. A few key questions remain unsolved regarding the assembly of the PWS-AS imprinted domain. In particular, it is unclear why *Ube3a* was fused to *SNRPN* and became a part of the PWS-AS imprinting domain, and no marsupial progenitors of *SNURF* and IC have yet been identified (Renfree et al., 2009).

## Pseudogenes of *Ube3a*

The two processed pseudogenes *Ube3ap1* and *Ube3ap2* have been identified in the human genome, although there is no evidence of their expression (Kishino and Wagstaff, 1998). These pseudogenes are also found in chimpanzees but not in mice and macaques, indicating that they formed in a common ancestor of chimpanzees and humans. *Ube3ap1* and *Ube3ap2* are located on chromosome 2 and 21, respectively, in the human and chimpanzee genomes.

## Conclusion and Perspective

*Ube3a* is an ancient gene that emerged prior to nervous systems, and its imprinted expression was acquired much later (Figure 2C). These findings accord with the current view that genes involved in human neurogenetic disorders are not necessarily evolutionarily new. To deepen the understanding of the evolution of *Ube3a*, comparison with the evolution of genes involved in brain disorders other than neurodevelopmental disorders, such as neurological disorders, is instructive. For example, presenilins (encoded by *PSEN1* and *PSEN2* in humans) form the catalytic center of γ-secretase that processes amyloid precursor protein (*APP*) to produce amyloid-β (Aβ) peptide, and mutations in *PSEN1*, *PSEN2* and *APP* are found in early-onset familial Alzheimer’s disease (Bertram et al., 2010). Presenilin orthologs are widespread among eukaryotes, including amoebozoans, metazoans and plants, suggesting that their ancestral gene was already present in the last common eukaryotic ancestor (Gazave et al., 2009). On the other hand, orthologs of the *APP* gene family (amyloid precursor-like protein 1 (*APLP1*), amyloid precursor-like protein 2 (*APLP2*), and *APP*) have been identified only in multicellular metazoans, including *Nematostella vectensis*, and the amyloidogenic Aβ motif and γ-secretase cleavage sites are conserved only across *APP* orthologs from jawed vertebrates (Tharp and Sarkar, 2013; Moore et al., 2014). Although phylogenetic studies of the proposed Ube3a substrates are yet to be conducted and searches for additional candidates of AS-relevant substrates should be continued, the evolutions of *Ube3a* and presenilin suggest that the ancient emergence of disease-related enzymes and more recent appearance of their relevant substrates could be a common evolutionary scheme of the key signal transduction components across different brain disorders.

Recent studies suggest that diverse symptoms of AS are mediated by distinct circuits, cell types, substrates and downstream pathways that act at different developmental stages (Mandel-Brehm et al., 2015; Silva-Santos et al., 2015; Judson et al., 2016). From an evolutionary point of view, it can be suggested that the key events in the evolutionary history of *Ube3a* led to the current etiology of AS. These events likely include: (1) expression of the Ube3a protein in nerve cells and its localization at functionally important subcellular compartments such as synapses; (2) colocalization and interaction with substrates that play essential roles in neuronal development and function; and (3) acquisition of genomic imprinting, leading to increased vulnerability of *Ube3a* to genetic damage. Thus, further studies of the expression and localization of Ube3a orthologs in primitive extant organisms, and exogenous expression and functional analyses of these orthologs in mammalian neurons, will broaden the evolutionary perspective of AS, as described for a few other synaptic proteins (Burkhardt et al., 2014; Yang et al., 2015).

## Author Contributions

MS wrote the manuscript.

## Funding

This work was supported in part by a grant from the Angelman Syndrome Foundation.

## Conflict of Interest Statement

The author declares that the research was conducted in the absence of any commercial or financial relationships that could be construed as a potential conflict of interest. The reviewer DT and handling Editor declared their shared affiliation, and the handling Editor states that the process nevertheless met the standards of a fair and objective review.
